# Mechanisms of tumor persistence in metastatic melanoma following successful immunotherapy

**DOI:** 10.64898/2025.12.11.692633

**Published:** 2025-12-15

**Authors:** Yingxiao Shi, Zoltan Maliga, Tuulia Vallius, Shishir M Pant, Roxanne Pelletier, Brigette Kobs, Solanky Priyanka, Yiwen He, Eliezer Van Allen, Sandro Santagata, Patrick Ott, Christine G Lian, Elizabeth I. Buchbinder, David Liu, Peter K Sorger

**Affiliations:** 1 Laboratory of Systems Pharmacology, Harvard Program in Therapeutic Science, Harvard Medical School, 200 Longwood Avenue, Boston, MA 02115; 2 Ludwig Center at Harvard; 3 Division of Population Sciences, Dana-Farber Cancer Institute, 450 Brookline Avenue, Boston, MA 02215; 4 Department of Immunology, Harvard Medical School; 5 Parker Institute for Cancer Immunotherapy, Dana-Farber Cancer Institute, Boston, MA; 6 Department of Pathology, Brigham and Women’s Hospital, Harvard Medical School, 75 Francis Street, Boston, MA 02115; 7 Department of Dermatology, Brigham and Women’s Hospital, Harvard Medical School, 75 Francis Street, Boston, MA 02115; 8 Department of Dermatology, Massachusetts General Hospital; 9 Department of Medical Oncology, Dana-Farber Cancer Institute, 450 Brookline Avenue, Boston, MA 02215; 10 Department of Pathology, Brigham and Women’s Hospital, Harvard Medical School, 75 Francis Street, Boston, MA 02115; 11 Department of Systems Biology, Harvard Medical School, 200 Longwood Avenue, Boston, MA 02115.

## Abstract

Persistent stable lesions represent a common but ambiguous outcome in melanoma patients receiving immune checkpoint inhibitors (ICIs). However, these lesions are infrequently removed and poorly characterized. Here, we perform in-depth multi-omics spatial profiling on persistent stable lesions from six ICI-treated patients. In one, the proportion of viable and proliferating tumor cells was similar to that of site-matched tumors from patients progressing during ICI. Extensive infiltration with cytotoxic T cells and a high level of programmed cell death were also observed. Some quiescent cancer cells were present, but this was not the dominant tumor state. A second stable lesion, while pathologically negative, also contained proliferative tumor nests with proximate immune cells. These findings provide evidence in patients for extended tumor mass dormancy in which cell death balances ongoing proliferation and further demonstrate that persistent stable lesions can be reservoirs of viable tumor cells with implications for clinical monitoring and management.

## INTRODUCTION

Immune checkpoint inhibitors (ICIs) have revolutionized the treatment of metastatic melanoma, with long-term studies documenting ten-year survival rates as high as 44–52%.(1,2) In a substantial subset of responsive patients (anecdotally ~20–40%),(3) tumors shrink on therapy but remain palpable or visible by radiological imaging for an extended period of time without evidence of growth or metastasis.(4) The clinical and biological interpretation of these seemingly dormant melanomas is unclear, and they are not routinely removed in the absence of other evidence of disease progression. Thus, even though long-term stable lesions are not uncommon post-ICI treatment, acquiring such specimens for research purposes has been difficult. However, a recent clinical study by Buchbinder et al.(5) was successful in obtaining biopsy or resection specimens from multiple persistent stable lesions, providing a unique resource for studying mechanisms of tumor dormancy post-ICI treatment.

Three nonexclusive mechanisms have been proposed to explain the perdurance of dormant tumors following treatment.(6) In angiogenic dormancy, a form of “tumor mass dormancy,” cancer cells continue to proliferate, but tumors remain constant in size as a result of a balance between proliferation and programmed cell death (PCD).(7) A similar phenomenon is observed in cultured tumor cells exposed to anti-cancer drugs: what appears to be population-level cytostasis (no net growth due to cell cycle arrest) is often a dynamic state with matched (or nearly matched) division and death rates.(8) Immunogenic tumor mass dormancy(9) is a related phenomenon in which surveillance by the adaptive immune system (T cells in particular) kills proliferating tumor cells in a rough balance with the numbers that are born.

Dormancy at a single cell level has attracted the greatest recent interest and involves tumor cells that enter a prolonged period of quiescence or senescence with little evidence of proliferation.(10,11) In animal models, quiescent cancer cells (QCCs) have been observed to occupy specialized niches in the tumor microenvironment (TME), and it has been proposed that this allows them to resist T cell attack,(12) potentially giving rise to long-lived micro metastases.(13) The creation of disseminated tumor cells (DTCs) is similar in that small clusters of QCCs are thought to contribute to residual disease and promote recurrence.(14) QCCs have been described in some studies as resembling stem-like tumor-initiating cells(15) that allow them to “seed” new tumor growth. However, other studies have found that cells capable of initiating new tumors are relatively common,(16) suggesting that QCCs might arise from the bulk tumor population via “phenotypic plasticity” and subsequent cell cycle exit. Phenotypic plasticity has also been observed outside of the setting of QCCs and is known to negatively impact responses to targeted and ICI therapy.(17)

The cohort identified by Buchbinder et al.(5) comprises seven patients in whom the existence of radiologically stable disease following ICI therapy provides a setting for studying features of dormant melanomas (specimens from six patients remain available).(5) These patients were treated with at least two doses of ipilimumab, nivolumab, or ipilimumab plus nivolumab and then followed by CT or PET/CT imaging at regular intervals. Tumors were scored as exhibiting a long-term stable state based on the absence of a steady increase in radiological signal for ≥ 6 months, with ICI therapy having first started > 2 years previously. Pretreatment and posttreatment biopsies and tissue specimens from this cohort were previously studied by hematoxylin and eosin (H&E) staining and standard immunofluorescence imaging; in this paper, we perform detailed multi-modal spatial profiling to identify the underlying mechanisms of tumor persistence and dormancy.

One lymph node metastasis in the cohort (specimen MEL101) was stable on CT imaging for 35 months but found to be FDG-avid on PET/CT and was therefore biopsied. It was subsequently resected based on evidence of viable tumor cells in the study biopsy. The resulting specimen is unique in being large enough for detailed analysis by spatial proteomics, spatial transcriptomics, and single cell RNA sequencing (scRNA-seq). Analysis of MEL101 revealed the co-occurrence of a high fraction of proliferating tumor cells, active T-cell mediated immune editing, expression of quiescence markers, and several types of PCD. The frequency of these states varied across the tumor, but in no case was quiescence the dominant phenotype. Moreover, none of the tumor cell states found in this tumor (e.g., dedifferentiated, neural crest-like, or melanocytic) were enriched in quiescent cells. We conclude that this exceptional post-ICI tumor was characterized by one or more forms of tumor mass dormancy rather than true quiescence. We also identified viable and proliferative tumor cells in another patient with a pathologically negative tumor (one judged to be scar-tissue based on H&E images), along with surrounding T cells and other immune cells. This is likely to represent another example of tumor mass dormancy rather than complete pathological response.

## RESULTS

**Figure 1.**
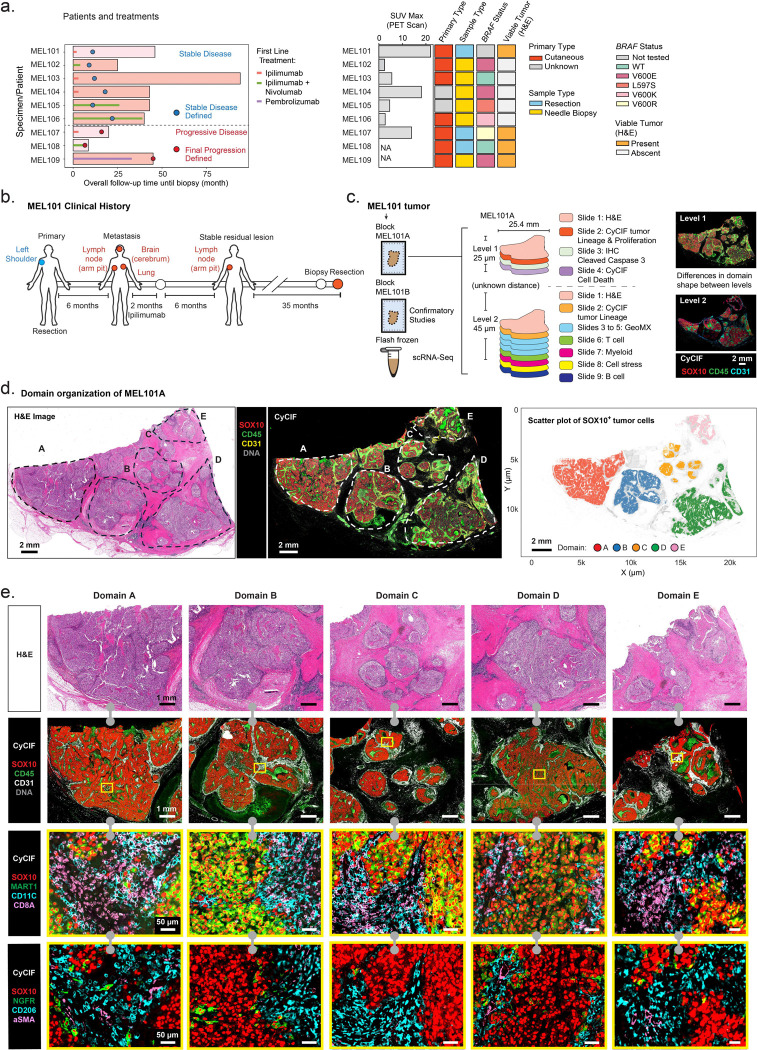
Multimodal profiling of patient MEL101 and the study cohort. **a.** Overview of the patient cohort summarizing the treatment history and clinical features of patients included in this study. **b.** Clinical timeline of patient MEL101. Patient MEL101 was initially diagnosed with a primary melanoma on the left shoulder, which was surgically resected. Six months later, metastatic lesions were detected in the axillary lymph node, cerebrum, and lung. The patient subsequently received two months of standard-dose ipilimumab treatment, which resulted in regression of all metastatic lesions except for the lymph node metastasis. This lesion was classified as a stable residual lesion six months after treatment. Serial ^18^FDG-PET/CT scans were performed every 3–6 months, and the residual lesion was surgically resected 35 months later. **c.** Schematic of tumor specimens and multimodal experimental approach. The MEL101 tumor yielded multiple tumor blocks and a frozen specimen used for single cell RNASeq. FFPE block MEL101A was used to generate multiple 5-micron sections in which Levels 1 and 2 refer to different regions of the same tumor separated along the Z-axis by an unknown amount of tumor removed from the block by other investigators. The tumor differed slightly in morphology from Levels 1 and 2, but the key features and five tumor domains were preserved. Block MEL101B was used in confirmatory studies to check overall tumor architecture. **d.** Overview of histopathological domains in specimen MEL101A. Domains A-E are indicated with dashed contours. *Left*: H&E-stained section; *Middle*: CyCIF image showing SOX10 (red), CD45 (green), CD31 (yellow), and DNA (grey); *Right*: scatter plot of SOX10^+^ tumor cells annotated by tumor domains. Scale bars, 2 mm. **e.** Magnified regions of annotated tumor domain A–E; grey barbell icons indicate that images are from the same or serial sections. *Top*: H&E-stained fields of view annotated with domain labels. *Bottom*: CyCIF images of adjacent sections showing staining for a variety of tumor and immune markers. *Row 2*: SOX10 (red), CD45 (green), CD31 (white), and DNA (grey). *Row 3*: SOX10 (red), MART1 (green), CD11C (cyan), and CD8A (pink). *Row 4*: SOX10 (red), NGFR (green), CD206 (cyan), and αSMA (pink). The regions outlined with yellow rectangles in *Row 2* represent the magnified regions shown in *Rows 3* and *4*. See Supplementary Table S3 for how markers were used to identify different cell types. Scale bars, 1 mm and 50 μm.

The Buchbinder et al.(5) melanoma cohort used in this study comprises specimens from metastatic melanoma patients treated with ICIs at least 2 years previously, in whom periodic CT or PET/CT imaging demonstrated the presence of stable melanoma lesions for a period of ≥ 6 months (see Supplementary Table 1 for clinical metadata). Biopsy or resection specimens of these lesions were available for six of these patients (MEL101 to 106), with their clinical course and disease characteristics summarized in Fig. 1a. As previously reported, only one of the six sampled lesions (MEL101), a lymph node metastasis, contained viable tumor cells detected by pathologist review of H&E-stained images. The other samples contained melanophages (macrophages that have ingested melanin) and necrotic or scar tissue but were scored as having no viable tumor cells. To compare persistent stable lesions to progressive metastatic melanoma, we acquired biopsies from three patients exhibiting progressive lymph node disease after ICI therapy (MEL107–109; Fig. 1a; Supplementary Fig. 1a, b). Freshly cut FFPE sections from these specimens were H&E-stained and subjected to expert pathology review to confirm tumor involvement (Fig. 1a). Adjacent sections were characterized by multiple spatial profiling methods, including cyclic immunofluorescence (CyCIF) imaging(18) and GeoMx microregional spatial transcriptomics. Profiling was performed with multiple CyCIF panels to enable discrimination of tumor differentiation states (e.g., SOX10, MART1, PMEL, and NGFR), proliferation (KI67, PCNA, and phospho-histone H3 (pH3)), apoptosis (cleaved caspase 3 (cCC3) and cPARP1), stromal structures (CD31 and αSMA), as well as immune lineages (e.g., CD45, CD3E, CD8A, CD4, FOXP3, CD20, CD11C, CD68, and CD163), and their activity states (e.g., PD1 and GZMB). Panels, abbreviations, and key properties of these markers are described in Supplementary Fig. 2a, b and Supplementary Table 2, 3.

MEL101, the largest specimen, was subjected to the most detailed spatial profiling; this lymph node metastasis was obtained from a patient who had a primary melanoma on the left shoulder that was treated with wide local excision. Six months later, the patient presented with metastases in the left axillary lymph node, brain, and lungs (Fig. 1b). This patient received four cycles of ipilimumab (anti-CTLA4) over two months, with an initial response observed in all lesions. Ultimately, all lesions regressed except for the left axillary lymph node metastasis that remained radiographically stable on CT for 35 months (Fig. 1b). Biopsy and H&E imaging revealed the presence of tumor cells and the lesion was therefore resected. Of the six patients with stable lesions biopsied, this was the only case in which tumor cells were detected via standard pathologic evaluation. Because of its size, this specimen provides a unique opportunity to perform detailed spatial profiling of a stable tumor. Thirteen 5 μm sections from two tissue blocks were therefore subjected to 30 to 64-plex CyCIF, 16-plex ORION imaging(19) (ORION is a one-shot multiplexed fluorescence imaging method that is particularly effective with fragile biopsy specimens), immunohistochemistry (IHC), and GeoMx whole-transcriptome-profiling (Fig. 1c; Supplementary Fig. 2c and Supplementary Table 2, 4). A total of 133 GeoMx microregions (MRs), each containing ~125–1500 cells and representing tumor and non-tumor regions were selected for characterization based on tissue morphology markers (Supplementary Fig. 2c–e) in CyCIF images of adjacent sections. Prior to mRNA preparation, some MRs were separated into tumor and immune compartments (this process is referred to as “segmentation” in the GeoMx workflow, but we refer to it as “enrichment” to avoid confusion with the conventional use of segmentation in image analysis). An adjacent fresh frozen tumor tissue from block 1 was used for scRNA-seq (Fig. 1c; Supplementary Fig. 2f, g).

**Figure 2.**
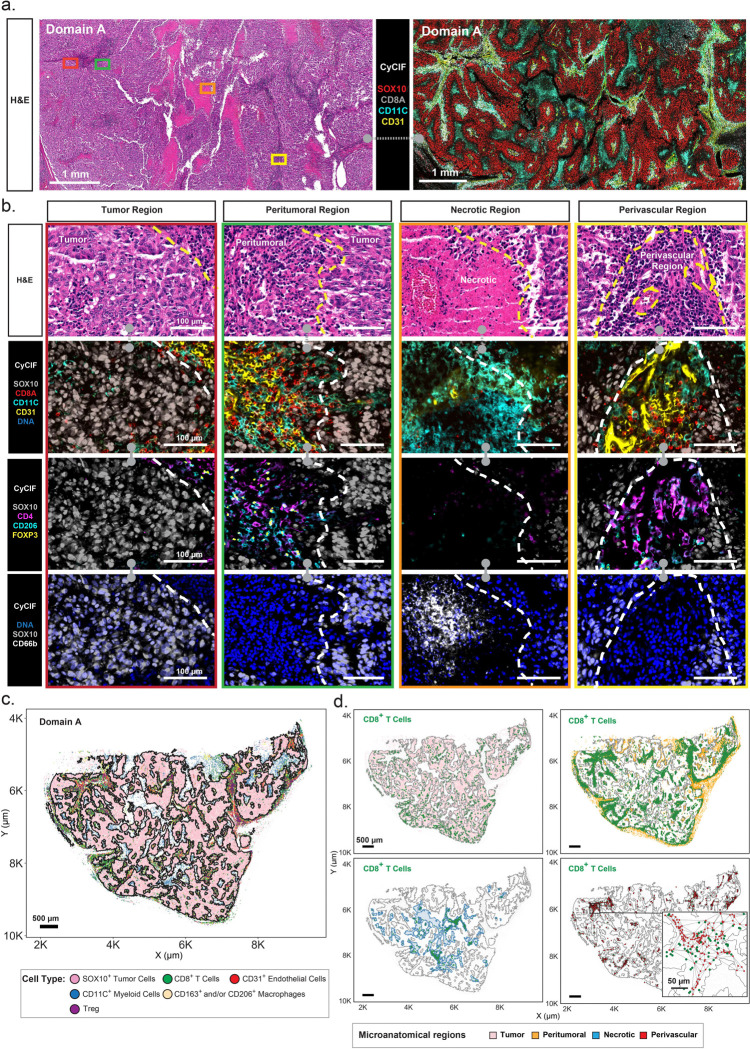
Characterization of MEL101 histopathological domains and microanatomical regions. For H&E-stained and CyCIF images, the grey barbell icon denotes the same CyCIF image with different channels shown; the barbell with dashed connector denotes adjacent sections. **a**. Field of view of MEL101 Domain A (sections 1 and 2 from MEL101A Level 1) showing H&E-stained image (left) and corresponding CyCIF image (right) with selected markers of a 64-plex image shown: SOX10 (red), CD8A (grey), CD11C (cyan), and CD31 (yellow). The regions outlined with rectangles represent the magnified regions shown in panel *b*. The color of each rectangle denotes a different microanatomical region: Tumor (red), Peritumoral (green), Necrotic (orange), and Perivascular (yellow). Scale bars, 1 mm. **b.** Magnification views of four representative microanatomical regions in Domain A (as depicted by colored boxes in panel *a*). From left to right: Tumor region, Peritumoral region, Necrotic region, and Perivascular region*. Row 1*: H&E-stained microanatomical regions. *Row 2* (CyCIF): SOX10 (grey), CD8A (red), CD11C (cyan), CD31 (yellow), and DNA (blue). *Row 3* (CyCIF): SOX10 (grey), CD4 (purple), CD206 (cyan), and FOXP3 (yellow). *Row 4* (CyCIF): SOX10 (grey), CD66 (white), and DNA (blue). Scale bars, 100 μm. The yellow and white dashed lines denote tumor boundary defined by SOX10^+^ tumor cells. **c.** Scatter plot displaying the spatial distribution of selected cell types within Domain A. The black outline denotes tumor boundary defined based on SOX10^+^ tumor cells. Each dot represents a single cell. Scale bar, 500 μm. **d.** Scatter plots showing the spatial distribution of CD8^+^ T cells (green) within the microanatomical regions: tumor (pink, top left), peritumoral (orange, top right), necrotic (blue, bottom left) and perivascular (red, bottom right) regions. The black rectangle indicates a magnified perivascular region with CD8^+^ T cells (green dots) and CD31^+^ cells (red dots). Black lines in the perivascular regions represent a Delaunay-based connectivity network between CD31^+^ cells. Scale bars, 500 μm and 50 μm.

Sections of MEL101A obtained at two levels of the tissue block (Levels 1&2) contained multiple distinct tumor and immune cell-rich domains (Fig. 1c), each of which was 1–8 mm in greatest dimension (~ 1–14 × 10^4^ cells each) separated by fibrotic tissue. Based on this overall structure we grouped the tumor into 5 separate domains (A–E) in Level 1 (dashed lines in Fig. 1d). The proportions of tumor and immune cells and the extent of necrosis and fibrosis varied among the five tumor domains with A being the most cellular and E the most fibrotic (Supplementary Fig. 3a–b). Each of the five domains contained a mixture of tumor, stromal and immune cells (Fig. 1e) that were found in microanatomical regions corresponding to tumor bed and peritumoral, necrotic, and perivascular regions (Fig. 2a, b; see METHODS). An adjacent specimen (MEL101B) had a similar organization bit with less intervening fibrotic tissue (Supplementary Fig. 2h). Averaged across the five tumor domains, ~58% of cells were SOX10^+^ tumor cells whereas 42% were lymphocytes, myeloid cells, and stromal cells of various types (Fig. 2b–d; Supplementary Fig. 3a). However, considering the specimen as a whole, the vast majority (95%) of lymphocytes and myeloid cells were found in peritumoral and perivascular regions rather than within the tumor bed itself (Fig. 2d; Supplementary Fig. 3c, d). Necrotic regions, which were particularly prominent within tumor Domain A, contained cellular debris of various types and frequently stained positive for the CD66B cell surface glycoprotein, which is consistent with tumor necrosis with neutrophilic response (Fig. 2b; Supplementary Fig. 3c, d – Necrotic region).

Expert pathology review of CyCIF and H&E data confirmed tumor necrosis, stromal fibrosis, and the presence of prominent immune infiltrates within the tumor bed; these are all characteristic features of immune-related tumor regression(20). Based on the presence of these histopathology features of established significance, we hypothesized that MEL101 Domains A–E correspond to different stages of a regression process. Domain A has the greatest level of active tumor killing and necrosis with neutrophilic and lymphocytic infiltration; Domain B shows continued immune infiltration with the emergence of early stromal fibrosis. Domain C and D, characterized by more prominent stromal fibrosis, likely represent later stages of immunoediting and tissue remodeling, while Domain E reflects the most advanced stage of fibrosis. We set out to test this hypothesis using molecular data from CyCIF and GeoMx.

### Tumor cell proliferation in persistent stable and progressing lesions

**Figure 3.**
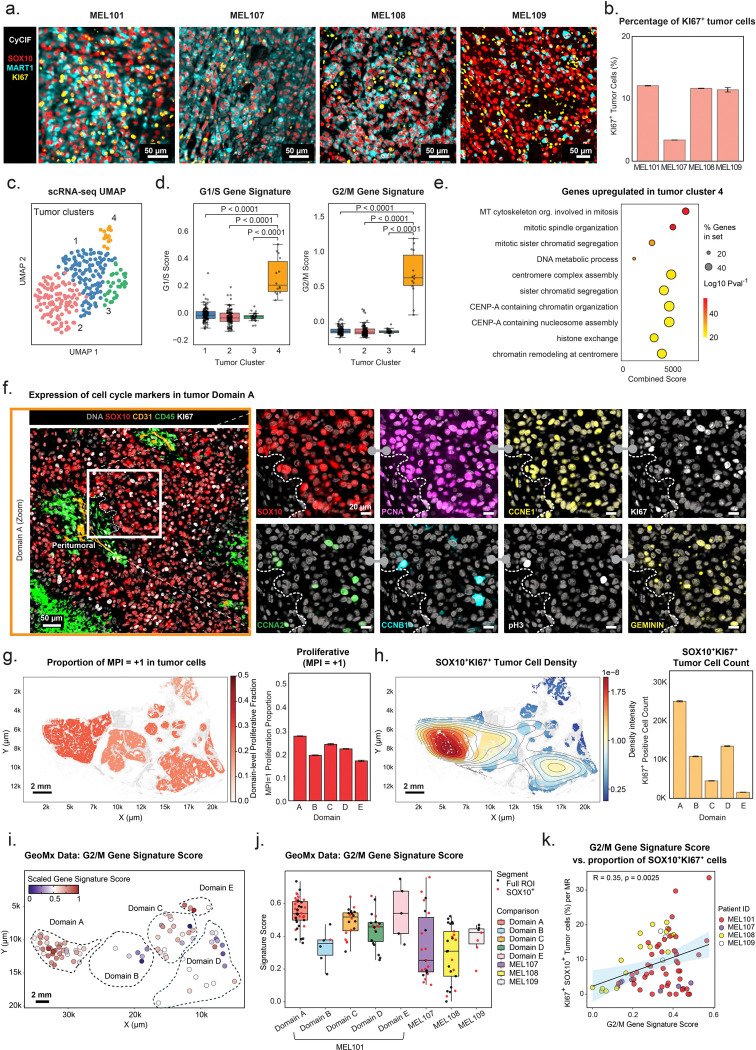
Tumor cell proliferation in persistent stable and progressing lesions. **a.** Magnified CyCIF images showing selected channels from a 42-plex CyCIF image: SOX10 (red), MART1 (cyan), and KI67 (yellow) within MEL101 and progressing tumors (MEL107-MEL109). Scale bars, 50 μm. **b.** Bar plot showing the percentage of SOX10^+^KI67^+^ tumor cells relative to all tumor cells in specimens MEL101 and MEL107–MEL109. Error bars represent binomial standard deviations. **c.** UMAP of tumor cells (scRNA-seq data) colored by tumor cell cluster ID. The clusters were obtained by unsupervised Leiden clustering. **d.** Boxplots comparing G1/S (left) and G/2M (right) gene signature scores across tumor clusters 1–4 from panel *c*. Boxes represent the first and third interquartile of the data; whiskers extend to show the rest of the distribution except for points identified as outliers. Statistical significance was assessed using the Mann–Whitney U test. **e.** Dot plot representing pathway enrichment analysis of genes upregulated in tumor cluster 4. Dot size reflects the proportion of enriched genes in each gene set, while color indicates statistical significance (−log_10_ *P*-value). The combined score represents the strength of enrichment for each pathway. **f.** Selected channels of a 64-plex CyCIF image from domain A of MEL101 showing cell cycle markers. *Left panel*: staining for DNA (grey), SOX10 (red), CD31 (orange), CD45 (green), and KI67 (white) within Domain A. The region outlined with a white rectangle represents the magnified region shown in the right panel; *Right panels*: magnified field of view stained for SOX10 (red), PCNA (purple), CCNE1 (yellow), KI67 (white), CCNA2 (green), CCNB1 (cyan), pH3 (white), and GEMININ (yellow). Grey barbell icon denotes the same CyCIF image with different channels shown. The white dashed lines denote tumor boundary defined by SOX10^+^ tumor cells. Scale bars, 50 μm and 20 μm. **g.** Spatial distribution and quantification of proliferative tumor cells (MPI = +1). *Left*: scatter plot of SOX10^+^ tumor cells in specimen MEL101A. Cells are color-coded by the average proportion of proliferative cells within each domain. Scale bar, 2 mm*. Right*: bar plot showing the proportion of MPI= +1 cells within each domain. Error bars represent binomial standard deviations. **h.** Spatial distribution and quantification of SOX10^+^ KI67^+^ proliferative tumor cells. *Left*: Kernel density estimation (KDE) plot highlighting the spatial density of SOX10^+^ KI67^+^ tumor cells across entire tumor. A Gaussian KDE was applied to the centroid coordinates of double-positive cells, with intensity clipped at the 98^th^ percentile and regions below the 10^th^ percentile masked for contrast enhancement. Contour lines denote high-density regions, overlaid on all cells (grey). The color scale represents kernel density-estimated spatial intensity (range: 0–1.8 × 10^−8^, scaled unit) *Right*: The absolute abundance of SOX10^+^KI67^+^ tumor cells across different domains. Scale bar, 2 mm. **i.** Scatter plot showing spatially resolved G2/M gene signature scores across GeoMx tumor MRs. Each data point represents one tumor MR, and color indicates the scaled gene signature score. Scale bar, 2mm. **j.** Boxplot comparing G2/M gene signature scores across tumor GeoMx MRs in Domains A–E and progressing tumors (MEL107–MEL109). Each dot represents a tumor MR (either SOX10^+^ segmented as part of the GeoMx RNA protocol or full MRs). Boxes represent the first and third interquartile of the data; whiskers extend to show the rest of the distribution except for points identified as outliers. **k.** Scatter plot showing the Pearson correlation between the G2/M gene signature scores (x-axis, GeoMx) and the percentage of SOX10^+^KI67^+^ tumor cells among all tumor cells (y-axis, CyCIF). Each data point represents an individual full tumor MR colored by patient ID. The solid line indicates the linear regression fit, with a 95% confidence interval shown (shaded area). Significance was calculated using a two-sided Pearson correlation test.

Analysis of CyCIF images showed that ~12% of tumor cells in MEL101 were positive for the KI67 proliferation marker (as were 35% of immune cells; Fig. 3a, b; Supplementary Fig. 4a); unexpectedly, this was similar to the average proportion of KI67^+^ tumor cells in progressing lymph node tumors in ICI-treated patients (MEL107–109; range 3% to 12% in Fig. 3a, b; Supplementary Fig. 4b). As further evidence that cells are actively dividing in MEL101, we scored G1/S, G2/M, and other signatures of cell division in scRNA-seq data and found these signatures to be enriched in a tumor cell cluster comprising ~5.4% of cells (cluster 4; Fig. 3c–e). We also analyzed tissue using a CyCIF panel that included markers specific to multiple stages of the cell cycle. The approximate order of expression (or appearance) of these markers in freely proliferating cells is as follows: the p21 and p27 cyclin dependent kinase (CDK) inhibitors are specific to cell cycle arrest and exit (p27 is also regarded as a tumor quiescence marker)(12), Cyclin D1 (CCND1) to G1/S cells, Cyclin E1 (CCNE1) and the DNA licensing factors PCNA and Geminin to S phase cells, Cyclin A2 (CCNA2) and Cyclin B1 (CCNB1) to G2/M cells, and phospho-histone H3 (pH3) to mitotic cells (Fig. 3f; Supplementary Fig. 4c). We found that adjacent tumor cells expressed these markers in various combinations consistent with their occupying different phases of the cell cycle, as expected for proliferating cells.

To integrate cell cycle data into a single multi-marker metric, we calculated the Multivariate Proliferation Index (MPI)(21) (Fig. 3g; Supplementary Fig. 4d, e). This confirmed the presence of proliferating tumor cells (MPI = +1) in all five tumor domains in roughly similar proportions (20–28% of tumor cells); GeoMx data also confirmed the enrichment of proliferation gene signatures in tumor MRs from all five domains. The greatest density of proliferating tumor cells (cells per unit area) was observed in Domain A (Fig. 3h). GeoMx gene signature scores were also higher in this than other MEL101 domains or in MEL107 to 109 (Fig. 3i, j; Supplementary Fig. 4f, g). This is expected since gene signature scores for an MR vary with the density of signature-positive cells within that MR, whereas the MPI proliferation index measures division on a per-cell basis. With this in mind, we found that CyCIF, scRNA-seq, and GeoMx data robustly and consistently demonstrate proliferation of tumor cells in MEL101, a clinically stable lesion, at levels comparable to tumor cell proliferation in lesions progressing on ICI therapy (Fig. 3k; Supplementary Fig. 4h).

### Immune surveillance of tumor domains in MEL101

**Figure 4.**
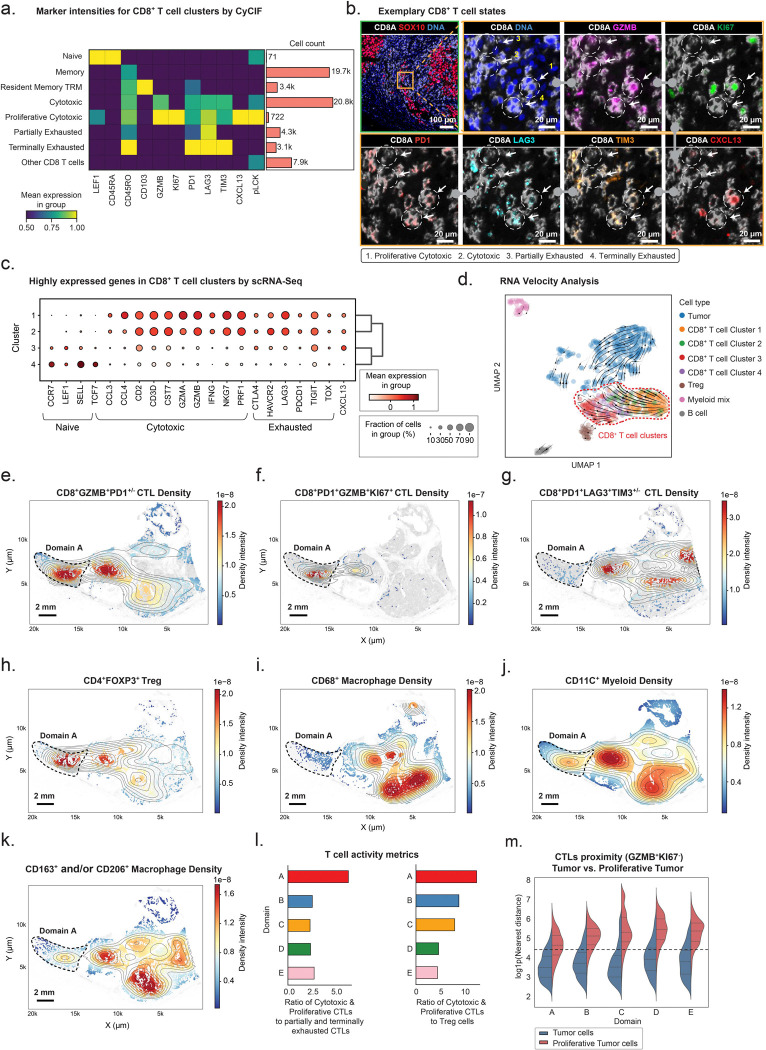
Immune surveillance of tumor domains in MEL101. **a.** Heatmap showing the mean scaled expression of selected CyCIF marker intensities across CD8^+^ T cell subtypes with assignment of their likely states: naïve, memory, tissue-resident memory, cytotoxic, proliferative cytotoxic, partially exhausted, terminally exhausted, and other. Bar plots on the right denote the absolute cell count in each subtype. **b.** CyCIF images of representative CD8^+^ T cell subtypes in a 41-plex CyCIF image showing staining for CD8A (white), SOX10 (red), DNA (blue), GZMB (magenta), KI67 (green), PD1 (red), LAG3 (cyan), TIM3 (orange), and CXCL13 (red). The white dashed circles and numbers highlight CD8^+^ T cell subtypes. Scale bars, 100 μm and 20 μm. **c.** Dot plot depicting expression of representative genes among CD8^+^ T cell clusters identified by Leiden clustering in scRNA-seq data. Dot size reflects the percentage of cells expressing each gene, while color intensity represents the average normalized expression level. Marker genes are grouped by functional categories: naïve, cytotoxic, and exhausted based on probable function. **d.** UMAP of scRNA-seq data colored by cell type. RNA velocity vectors overlaid on the UMAP illustrate dynamic state transitions, with a focus on the CD8^+^ T cell compartment (highlighted with a red dashed line) **e-k.** KDE plots showing spatial distribution of specific immune cell types across MEL101 domains. Contour lines indicate regions of high local density, overlaid on all cells (grey). The color scale represents kernel density-estimated spatial intensity (range in scaled units as shown). The black dashed region outlines Domain A. Scale bars, 2 mm. **e.** CD8A^+^GZMB^+^ CTLs with PD1 status undefined. **f.** Proliferative cytotoxic CD8^+^GZMB^+^KI67^+^ CTLs cells. **g.** Partially or terminally exhausted CD8A^+^LAG3^+^ and/or TIM3^+^ CTLs. **h**. CD4^+^FOXP3^+^ Treg cells. **i.** CD68^+^ macrophages. **j.** CD11C^+^ myeloid cells. **k**. CD163^+^ and/or CD206^+^ macrophages. **l.** Bar plots showing the ratios of cytotoxic (GZMB^+^) and/or proliferative (KI67^+^) CTLs relative to partially and/or terminally exhausted CTLs (left panel) or Tregs (right panel) across Domains A–E. **m.** Split violin plots of the distribution of log-transformed nearest-neighbor distances between cytotoxic CTLs (CD8^+^GZMB^+^KI67^−^) and either non-proliferative (blue) or proliferative tumor cells (red) in each domain.

The proportion of CD45^+^ immune cells in MEL101 tumor domains varied from 12–40%, demonstrating extensive infiltration (Supplementary Fig. 3a). Hierarchical cell type calling of CyCIF data identified seven CD8^+^ T cell states: (1) naïve T cells that co-expressed LEF1 and CD45RA, (2) memory T cells that expressed CD45RO, (3) resident memory T cells that co-expressed CD45RO and CD103, (4) cytotoxic T cells (CTLs) that expressed the cytolytic serine protease granzyme B (GZMB) as well as the PD1, LAG3, and TIM3 checkpoint proteins at intermediate levels, (5) CTLs similar to (4) but also expressing KI67, CXCL13, and pLCK (Tyr 394), a tyrosine kinase associated with the T cell receptor (TCR), (6) partially exhausted T cells that co-expressed PD1 and LAG3 with little or no TIM3, and (7) terminally exhausted T cells that co-expressed PD1, LAG3, and TIM3 (Fig. 4a, b; Supplementary Fig. 5a). The proportions of these cell types (relative to immune cells as a whole) differed by ~200-fold, with CTLs (Cytotoxic and Proliferative Cytotoxic, n = 21.5k) and memory T cells (Memory and Resident Memory, n = 23.1k) the most common and naïve T cells the least (cluster 1; n = 71). Naïve T cells were primarily localized to one organized lymphoid structure while other T cell subtypes were distributed across the tumor domains (Supplementary Fig. 5d–f; Fig. 4e–g).

scRNA-seq analysis of *CD3E*^+^*CD8A*^+^ cells (n = 344) confirmed CD8^+^ T cell diversity. Unsupervised Leiden clustering of scRNA-seq data generated four major CD8^+^ T cell clusters (Fig. 4c and Supplementary Fig. 5b, c): Cluster 2 cells expressed the *CCL5* chemokine, pore-forming protein perforin (*PRF1*), and *GZMB* at high levels and corresponded to CTLs; Cluster 1 cells expressed the *HLA-DR* and granzyme A (*GZMA*), and corresponded to a second population of CTLs; Cluster 4 cells expressed genes found in naïve and memory T cells such as *SELL, KLF2* and *TCF7*; and Cluster 3 cells expressed high level of *CXCL13* and the *PDE3B* phosphodiesterase and likely corresponded to the CXCL13 positive CTLs identified by CyCIF. RNA velocity analysis on these data yielded a trajectory from naive CD8^+^ T cells (Cluster 4) to *CXCL13*^+^*CD8*^+^ T cells (Cluster 3) to cytotoxic states (Clusters 1 and 2), consistent with an active T cell response (Fig. 4d) involving naïve, cytotoxic, and activated/exhausted T cells in proportions consistent with ongoing tumor immunoediting.

The relative abundance of T cell subtypes varied substantially with tumor domain (Fig. 4e–g; Supplementary Fig. 4d–f). Proliferative CXCL13^+^ CTLs were enriched in Domain A (Fig. 4f), which also contained the highest density of proliferating tumor cells (Fig. 3g–h). Tregs, macrophages and myeloid cells were present across all domains (Fig. 4h–k) with Tregs enriched in Domain A (Fig. 4h); macrophages (Fig. 4i, k) and myeloid cells (Fig. 4j) enriched in Domains C–E. Proximity analysis showed that CTLs were closest to proliferative tumor cells in Domain A as compared to other domains (Fig. 4m). Moreover, the ratio of CTLs (and proliferative CTLs) to exhausted T cells was highest in Domain A (Fig. 4l). These are all features of an active anti-tumor immune response and consistent with our hypothesis that Domain A represents an earlier and more active stage of immune editing than Domain B, which has a higher proportion of exhausted T cells, or Domains C–E, which have few CTLs and more abundant macrophages and fibrosis, consistent with immune downregulation and resolution.

### Programmed cell death in tumor compartments

**Figure 5.**
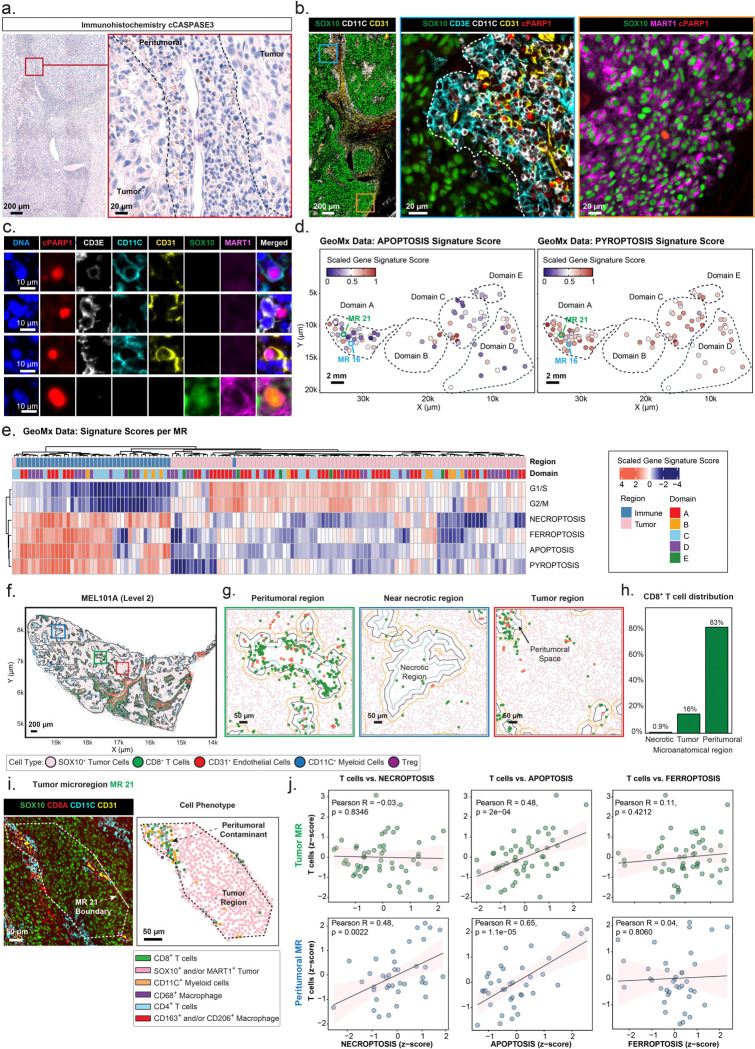
Programmed cell death in the tumor compartment in MEL101. **a.** IHC image of cleaved caspase-3 (cCASPASE3) staining within MEL101 Domain A (Level 2). The black dashed line separates the tumor and peritumoral regions. Scale bars, 200 μm and 20 μm. **b.** *Left*: selected channels from a 42-plex CyCIF showing staining for SOX10 (green), CD11C (white), CD31 (yellow). The regions outlined with blue and orange rectangles represent the magnified regions shown in the middle and right panels, respectively; *Middle*: magnified region highlighting cPARP1^+^ non-tumor cells within the peritumoral region: SOX10 (green), CD3E (cyan), CD11C (white), CD31 (yellow), and cPARP1 (red). White dashed line denotes tumor boundary defined by SOX10^+^ tumor cells; *Right*: magnified region highlighting a cPARP1^+^ tumor cell within the tumor region: SOX10 (green), MART1 (magenta), and cPARP1 (red). Scale bars: 200 μm and 20 μm. **c.** Selected channels from a 43-plex CyCIF image showing cPARP1^+^ cells: DNA (blue), cPARP1 (red), CD3E (white), CD11C (cyan), CD31 (yellow), SOX10 (green), and MART1 (magenta). Scale bar, 10 μm. **d.** Scatter plot showing spatially resolved APOPTOSIS (left) and PYROPTOSIS (right) signature scores across GeoMx tumor MRs. Each data point represents a tumor MR, and color indicates the scaled gene signature score. Scale bars, 2 mm. **e.** Heatmap displaying gene signature scores for programmed cell death and cell cycle signatures across annotated tumor Domains A–E. Columns represent individual GeoMx MRs, annotated by segment type (immune vs. tumor) and domain identity. **f–g.** Scatter plots showing the distribution of SOX10^+^ tumor cells (pink), CD8^+^ T cells (green), CD31^+^ endothelial cells (red), CD11C^+^ myeloid cells (blue), and FOXP3^+^ Treg (purple) within Domain A from MEL101A Level 2. **f.** Entirety of tumor Domain A. The regions outlined with rectangles represent the magnified regions shown in panel *g*. The colors of the rectangles denote different microanatomical regions: Peritumoral (green), Necrotic (blue), and Tumor (red). The black outline denotes tumor boundary defined by SOX10^+^ tumor cells. Scale bar, 200 μm. **g.** Magnified views of Peritumoral, Necrotic, and Tumor regions from panel *f*, showing variation in CD8^+^ T cells and CD31^+^ endothelial cell densities across typical microanatomical regions. Orange, black and blue contours represent tumor margins. Scale bars, 50 μm. **h.** Bar plot quantifying CD8^+^ T cell abundance across microanatomical regions. **i.** Selected channels of 43-plex CyCIF images (left) showing the spatial composition of tumor and immune cell subsets within tumor MR 21 with SOX10 (green), CD8 (red), CD11C (cyan); Scatter plot (right) showing positions of selected tumor and immune cell types within GeoMx tumor MR 21: SOX10^+^ and/or MART1^+^ tumor cells (pink), CD8^+^ T cells (green), CD4^+^ T cells (blue), CD11C^+^ myeloid cells (orange), CD163^+^ and/or CD206^+^ macrophages (red), and CD68^+^ macrophages (purple). Scale bars, 50 μm. **j.** Scatter plots showing the Pearson correlation between NECROPTOSIS (left), APOPTOSIS (middle), and FERROPTOSIS (right) gene signature scores (x-axis) and T cells signature scores (y-axis) for tumor (top row) and peritumoral (bottom row) MRs. Each data point represents an individual tumor MR. The solid line denotes the linear regression fit with a 95% confidence interval (shaded area). Significance was calculated using a two-sided Pearson correlation test.

A tumor that is actively proliferating yet remains constant in size must experience ongoing cell death and clearance. Based on the prevalence of CTLs in MEL101, we anticipated a role for immune-mediated PCD, with the possible addition of cell-autonomous cell death arising from DNA damage, metabolic stress, or intrinsic defects in tumor cells. To visualize dying cells, we performed IHC using antibodies against cleaved caspase-3 (cC3), the active form of the executioner caspase in apoptotic cells, as well as CyCIF using antibodies against cleaved PARP1 (cPARP1), a downstream substrate of cC3 during caspase-dependent apoptosis (CyCIF and GeoMx were performed on MEL101A Level 2 – see Fig. 1c). Both methods identified dying cells in tumor and peritumoral microanatomical regions (Fig. 5a, b; Supplementary Fig. 6a, b). The majority of cPARP1-positive cells were non-tumor cells (e.g., CD3E^+^, CD11C^+^, or CD31^+^), particularly in peritumoral regions, consistent with the short half-lives of activated T cells, neutrophils and other immune cells (Fig. 5b, c). However, it was possible to find some cPARP1-positive tumor cells (Fig. 5c – 4^**th**^ **row**, representing < 1% of all SOX10^+^ cells).

As a complementary approach, we analyzed GeoMx transcriptomic data using gene sets associated with different forms of PCD (see METHODS) and observed enrichment for necroptosis, pyroptosis, apoptosis, and ferroptosis with substantial variation across tumor domains (Fig. 5d; Supplementary Fig. 6c). Pyroptosis(22) and necroptosis(23) are immunogenic forms of PCD that generate an inflammatory environment and recruit immune cells, and pyroptosis has been associated with better outcomes in metastatic melanoma.(24) In contrast, neither apoptosis nor ferroptosis(25) is immunogenic, but both can be triggered in a cell-autonomous and non-autonomous manner. Many genes are common to the four PCD signatures and it is not possible to fully discriminate among them based on gene signatures alone. However, variation in the strengths of the PCD signatures across tumor domains (Fig. 5d, e; Supplementary Fig. 6c) suggested that cells in MEL101 were dying via multiple PCD pathways.

Dying cells were most numerous in peritumoral regions (Fig. 5e), which were also where >80% of all T cells were located (Fig. 5f–h). The large numbers of dying immune cells posed a challenge for the detection of PCD in tumor cells since signals from dying tumor and immune cells were likely to be intermixed. Moreover, it was not always possible to fully separate tumor and peritumoral regions in space during GeoMx imaging, due to the irregular and intertwined boundaries of these regions (Fig. 5i; Supplementary Fig. 6d). Nonetheless, when we compared the strengths of necroptosis and T cells signatures(26) across tumor MRs (Fig. 5j; Supplementary Fig. 6e), we observed a non-significant correlation in tumor MRs and significant correlation in peritumoral MRs (Fig. 5j – *NECROPTOSIS*). We interpret this as reflecting a significant level of necroptosis in the tumor region that is not immune mediated. In the case of apoptosis, a correlation was observed in both regions, possibly due to a strong contribution from dying T cells (Fig. 5j – *APOPTOSIS*). Ferroptosis represented a case in which a significant correlation in PCD and T cells signatures was not observed in either tumor or peritumoral regions (Fig. 5j – *FERROPTOSIS*). From these data, we conclude that PCD is ongoing in regions of MEL101 where immune cells are abundant (peritumoral regions) and also in the infiltrated tumor regions, and that the extent to which PCD arises from death of immune cells, T-cell mediated immunosurveillance, and intrinsic tumor cell death varies with the form of cell death and the domain or microanatomical region. PCD via ferroptosis and necroptosis seemed most likely to reflect intrinsic death in the tumor region. More generally, high levels of cell death are a key feature of the tumor mass dormancy model.

### Non-proliferative and quiescent cancer cell states

**Figure 6.**
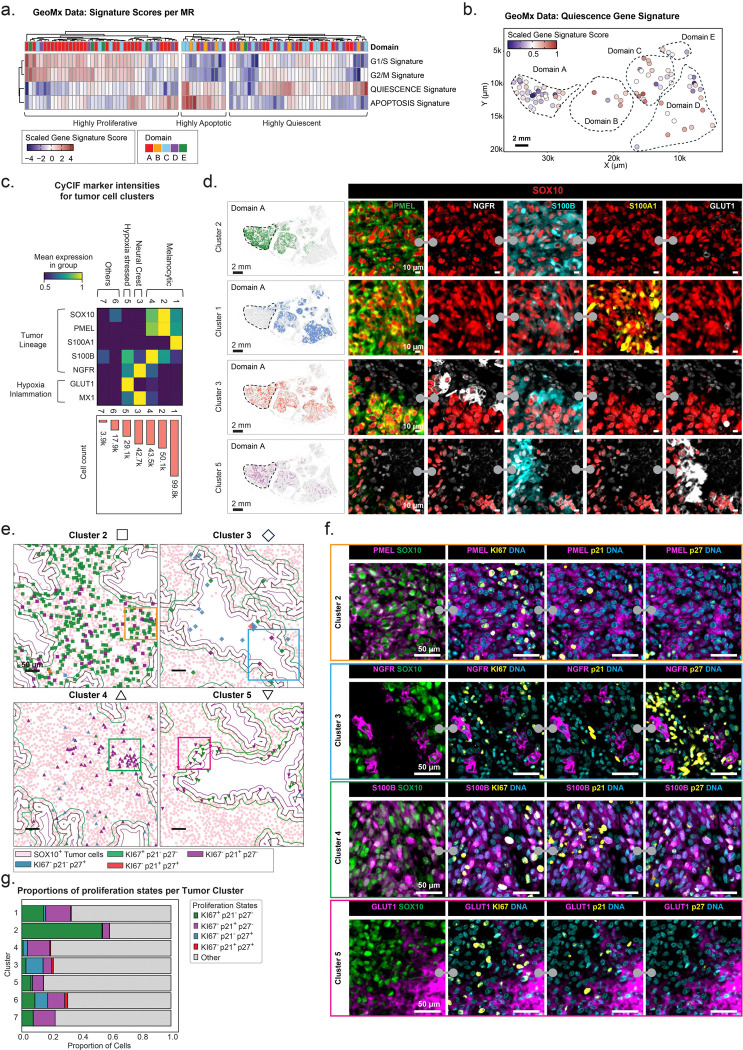
Presence of non-proliferative and quiescent cancer cell states in MEL101. **a.** Heatmap showing gene signature scores of gene signatures associated with proliferation, programmed cell death, and quiescence across all tumor MRs. Hierarchical clustering identified three dominant tumor states: highly proliferative, highly apoptotic, and highly quiescent. Columns represent tumor MRs annotated by Domains A–E (see color code below). **b.** Scatter plot showing spatially resolved QUIESCENCE signature scores across GeoMx tumor MRs. Each data point represents a tumor MR, and color indicates the scaled gene signature score. Scale bar, 2mm. **c.** Heatmap depicting the mean scaled expression of selected CyCIF markers for SOX10^+^ tumor clusters defined by Leiden clustering. The bar plot shows the absolute cell count for each cluster. **d.** Distributions of tumor cell states based on the clusters shown in panel *c*. *Left*: scatter plots depicting the spatial location of different tumor clusters, colored by cluster label, with background cells shown in grey. The black dashed region indicates Domain A. *Right*: selected channels from a 64-plex CyCIF image of regions of Domain A: SOX10 (red), PMEL (green), NGFR (white), S100B (cyan), S100A1 (yellow), and GLUT1 (white). Scale bars, 10 μm. **e.** Scatter plots of selected regions of Domain A showing the proliferation status of tumor cells in clusters 2–5. Each datapoint represents a tumor cell, shapes denote the clusters and color indicates the proliferation status as determined by p21 and p27 staining. The green, black and purple contour lines denote tumor margin. The regions outlined with colored rectangles are shown as CyCIF images in panel *f*. **f.** Selected channels from a 64-plex CyCIF images stained for SOX10 (green), PMEL (magenta), NGFR (magenta), S100B (magenta), GLUT1 (magenta), DNA (cyan), KI67 (yellow), p21 (yellow), and p27 (yellow), Scale bars, 50 μm. **g.** Stacked bar plot showing the proportions of KI67^+^p21^−^p27^−^ (green), KI67^−^p21^+^p27^−^ (purple), KI67^−^p21^−^p27^+^ (blue), KI67^−^p21^+^p27^+^ (red), and Other (grey) cells within each tumor cluster.

To explore the role of QCCs in MEL101, we quantified tumor cells that were negative for KI67 and positive for either or both of p21 (CDKN1A) and p27 (CDKN1B). By this definition, we found that QCCs represented 12% of all SOX10^+^ tumor cells, with the proportion varying from one tumor domain to the next. This is consistent with the presence of an MPI = −1 state in approximately 20% of SOX10^+^ tumor cells (Supplementary Fig. 7a; Supplementary Fig. 4e). We also performed gene signature analysis on both GeoMx and scRNA-seq data using quiescence gene signature(27) and found that the magnitude of this signature was inversely correlated with that of proliferation signatures (G1/S and G2/M) (Fig. 6a, b; Supplementary Fig. 7b, c). The strength of quiescence signatures varied within MEL101 domains but was overall comparable to signature scores observed in progressing tumors MEL107–109 (Supplementary Fig. 7d). Thus, both assessments of cell cycle marker levels (measured by CyCIF) or gene signatures (inferred from GeoMx data) demonstrate that QCCs are present in MEL101 but are less abundant than proliferating tumor cells.

To determine if specific tumor lineages were enriched in QCCs, we performed unsupervised clustering of CyCIF markers (for all SOX10 and/or PMEL-positive cells, Fig. 6c; Supplementary Fig. 7e). This resulted in seven tumor cell clusters; cells in these clusters had differing prevalences across MEL101 tumor domains (Supplementary Fig. 8b). Clusters 1, 2, and 4 represented melanocytic states, characterized by the highest levels of SOX10 and PMEL expression (Fig. 6c; Supplementary Fig. 7e) but with differences in the expression of S100 proteins: clusters 2 and 4 were S100B-high and predominantly located in tumor Domains A and B, while cluster 1 was S100A1-high and most prevalent in other domains (Fig. 6d, e; Supplementary Fig. 7f). The S100 family has 21 members that are expressed at varying levels across most solid tumors(28); expression of S100B is elevated in nevus cells relative to malignant melanoma, and the opposite is true of S100A1(29). The functional significance of these differences remains unclear, but differential expression of S100 proteins can be an effective means of identifying spatially patterned differences in melanoma cell states(29). Cells in tumor cluster 3 had elevated NGFR expression, consistent with dedifferentiation into a neural crest-like state (Fig. 6c, d); these cells were mainly found in peritumoral, immune cell-dense regions (Fig. 6d). Cells in cluster 5 had lower expression of SOX10 and PMEL but high expression of the GLUT1 glucose transporter, NQO1 (NAD(P)H quinone oxidoreductase 1), and COX4 (a mitochondrial cytochrome c oxidase); all three proteins are associated with a metabolically active state and in some cases metastasis and poor prognosis (Fig. 6c, d).(30,31) These cells were primarily found adjacent to necrotic regions, which represents a hypoxic environment sometimes associated with increased cancer aggressiveness (Fig. 6e, f).(32)

These data demonstrate the presence of spatially distinct melanoma lineages and microenvironments across MEL101. However, all lineages and local environments that we have been able to identify comprised a mix of dividing and arrested cells with no evidence that quiescence was restricted to specific tumor states or spatial patterns (Fig. 6f, g). In particular, p27 positive and MPI = −1 cells were not obviously restricted to perinecrotic, perivascular, or other discernable niches in the tumor compartment. Thus, the prevalence and spatial distribution of p27-high and MPI = −1 QCC is insufficient to explain the maintenance of a tumor dormancy.

### Proliferating tumor cells and immune surveillance in a stable lesion involving scar tissue

**Figure 7.**
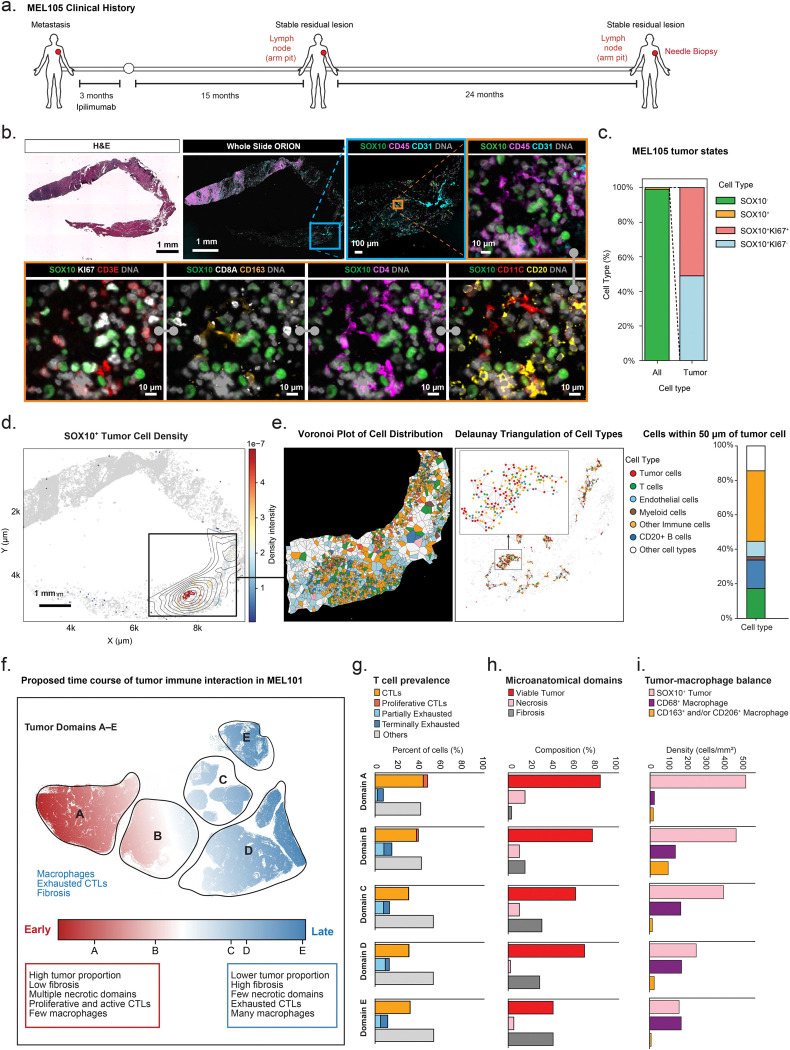
Proliferating tumor cells and immune surveillance in a stable lesion involving scar tissue. **a.** Clinical timeline for patient MEL105, whose tumor was biopsied after 24 months of stable disease. **b**. H&E-stained and ORION images of the needle biopsy specimen from MEL105. The grey barbell icon denotes the same ORION image with different channels shown. Selected channels from an 18-plex ORION images shown: SOX10 (green), CD45 (magenta), CD31 (cyan), DNA (grey), KI67 (white), CD3E (red), CD8A (white), CD163 (orange), CD4 (magenta), CD11C (red), CD20 (yellow). The regions outlined with blue and orange rectangles represent progressively magnified regions. Scale bars, 1 mm, 100 μm, and 10 μm. **c.** Stacked bar plot showing the percentage of tumor cells relative to all cells in the specimen (~1%) and the proliferation status of these cells in specimen MEL105. **d.** KDE plot showing spatial distribution of SOX10^+^ tumor cells in specimen MEL105. Contour lines indicate regions of high local density, overlaid on all cells (grey). The color scale represents kernel density-estimated spatial intensity (range: 0–5 × 10^−7^, scaled unit). The region outlined with the black rectangle represents the magnified region in panel *e*. Scale bar, 1mm. **e.** *Left*: Voronoi plot showing the distribution of tumor, immune, and stromal cell types within the highlighted area in panel *d*. *Middle*: Delaunay triangulation plot of cell types surrounding SOX10^+^ tumor cells within a 50 μm radius. Black lines represent a Delaunay-based connectivity network between nearby SOX10^+^ tumor cells and surrounding immune cells. The region outlined with the black rectangle represents the magnified region. Each dot represents a single cell, colored by cell type. *Right*: stacked bar plot showing the percentage of cell types within a 50 μm radius of SOX10^+^ tumor cells; color codes match those in the Delaunay triangulation. **f.** Schematic representation of tumor-immune interactions across Domains A–E. **g.** Stacked bar plots showing the proportion of CD8^+^ T cell subsets out of all CD8^+^ T cells within Domains A–E. **h.** Bar plots showing the histopathologic composition of Domains A–E, as evaluated from H&E images. **i.** Bar plots showing the density (cells/mm^2^) of SOX10^+^ tumor cells, CD68^+^ macrophages and CD163^+^ and/or CD206^+^ macrophages across Domains A–E.

ORION images of biopsies from the five additional specimens in the Buchbinder et al.(5) cohort for which tissue was still available (MEL102 to MEL106; Fig. 1a) revealed the presence of SOX10^+^ tumor cells in specimen MEL105 but no other specimens; MEL106 contained only fibrotic tissue, and the other biopsy specimens contained fibrotic tissue with infiltrating Treg and CD8^+^ T cells, CD163^+^ macrophages, CD20^+^ B cells, and CD11C^+^ myeloid cells, (Supplementary Fig. 8a). MEL105 was scored previously as a complete pathological response based on inspection of H&E images(5), but we identified 406 SOX10^+^ tumor cells out of a total of 40,670 segmented cells in the whole-slide image of the biopsy specimen (Fig. 7a, b). Remarkably, half of these tumor cells were KI67^+^, representing a higher proliferation index than observed in MEL101 or progressing tumors (Fig. 7c). Viable tumor cells were present in nests of 200–400 cells surrounded by diverse types of T and B cells, suggestive of active immune editing (Fig. 7d, e). We conclude that MEL105 is likely to represent another case of tumor mass dormancy with ongoing tumor cell proliferation counterbalanced by immune editing.

### Spatial variation in proliferation, death and dormancy

Variation in tumor and immune state across MEL101 is conceptualized in Fig. 7f as representing different phases of tumor-immune interaction. Domain A had the highest tumor and proliferating tumor cell density, in conjunction with the highest proportion of CTLs and proliferative CTLs, and highest level of necrosis (Fig. 7g–i). Domains B and C also had infiltrating CTLs, but also increasing proportions of macrophages, consistent with later stages of immune-mediated clearance of dead cells. Domains D and E were enriched in fibrosis, the terminal stage of tumor cell clearance. Thus, tumor mass dormancy in a single clinically stable tumor lesion is consistent with qualitatively distinct tumor states and phases of tumor-immune interaction.

## DISCUSSION

In this paper we use multimodal spatial profiling and conventional histology to study six metastatic melanomas that exhibited persistent residual (stable) radiographic disease following anti-CTLA4 and/or anti-PD1 directed immunotherapy. We found that two of them contained actively dividing tumor cells but also infiltrating CTLs, consistent with active T-cell mediated tumor surveillance. One stable tumor (MEL101) was sufficiently large that it provided a unique opportunity to study tumor mass dormancy in detail in a human patient rather than a mouse model.(33) Deep characterization of this tumor identified that the proportion of p27^+^ and/or p21^+^ QCCs in these tumors was similar to, or lower than the proportion of proliferating cells. This demonstrates that the persistent tumors we examined are not primarily comprised of quiescent (non-dividing) cancer cells but have instead achieved a state of tumor mass dormancy involving ongoing cancer cell proliferation and balanced cell death. We found no evidence of tumor cells in the other four specimens, but biopsies contained viable immune cells, and it is likely that these contributed to the FDG signal detected on PET.

The observation that cells in MEL101 are as likely to express proliferation markers as tumors progressing on ICI therapy is striking. At a macroscopic level, the presence of necrosis, particular in tumor Domain A, is *prima facie* evidence of ongoing cell death, most likely of both tumor and immune cells. Necrosis is often considered to be a negative prognostic feature(34,35), but in MEL101 it is clearly compatible with effective immune-mediated tumor control. We were able to detect both individual dying tumor cells and fields of extensive death by CyCIF and to infer the activation of four different PCD pathways by GeoMx and scRNA-seq data. However, clearly distinguishing death of immune cells from immune-mediated killing of tumor cells is challenging in the crowded environment of an infiltrated tumor. The high level of CTLs in MEL101 nonetheless argues that much of the tumor cell death we observe is immune-mediated and suggests that once activated by ICIs, anti-tumor immunity can persist for years. Our data also suggest the involvement of cell-intrinsic PCD, potentially due to cell stress and metabolic deficiencies, but this is difficult to conclusively prove in the background of immune-mediated PCD.

Recent research on persistent tumors in model organisms has focused on tumor cell quiescence and the factors that promote the QCC state. We detected cells with the properties of QCCs throughout MEL101, but these were not enriched in any previously described tumor states (e.g., dedifferentiated, neural crest-like, or melanocytic). Moreover, a p21 and/or p27 high QCC state was not the dominant tumor cell state, and it was not patterned spatially. This is suggestive of exit and entrance of cells from proliferation - a normal feature of cell division - rather than wide-spread entry into a dormant, quiescent, or senescent state. Our results therefore support earlier theories of tumor mass dormancy involving a dynamic balance between tumor cell division and cell death(7).

Understanding properties of dormant tumors and the sites they occur is of considerable clinical and biological interest since dormancy may be widespread. A long-term study of thin primary melanomas (T1) showed that 15–29% of patients died from melanoma at 20 years after original diagnosis, with the majority of deaths occurring >5 years after diagnosis(36). Clinical evidence that the immune system plays a role in tumor dormancy is based primarily on transplantation cases in which immunosuppressed recipient patients developed melanoma after receiving organs from an immunocompetent donor who had a history of melanoma that was completely resected without evidence of existing disease.(37,38) Other evidence of tumor-immune interactions leading to tumor dormancy have come primarily from pre-clinical models(9,39). In these models, the immune system is observed to have a cytostatic effect on the tumor, resulting in a decrease in markers of tumor proliferation in the dormant vs progressive tumors. In contrast, our data reveal tumor proliferation in persistent stable lesions at the same or higher level than melanomas from patients with progressing lymph node tumors.

The dynamics of tumor mass dormancy within a single patient’s tumor are strikingly diverse and more varied that what has been observed in preclinical models. Within a single tumor, different regions display variable degrees of proliferating tumor cell density, immune cell activation, and cell death, all within a setting of thick fibrosis with no evidence of residual normal lymph node architecture (Fig. 7f). This suggests dynamisms in the tumor-immune interaction, with regions of increased active tumor proliferation and anti-tumor immune response, co-existing with other relatively quieter regions. Fibrosis likely represents scars of past tumor-immune battles whereas necrosis is the more immediate sequela of tumor and immune cell death. Across MEL101, necrosis is most prominent where immune cell killing is greatest (in Domain A) and fibrosis where CTLs are least abundant, and macrophages enriched. Determining whether this reflects different stages of an anti-tumor immune response (as hypothesized in Fig. 7f) or differences in tumor clones is an important direction for future inquiry.

From a clinical perspective there is robust data that ICIs can provide long-term disease control in a substantial subset of advanced melanoma patients,(2,40) but late progression (>5 year) in ICI-treated advanced melanomas has been reported to occur in 10–50% of responders depending on the setting and criteria.(41,42) Thus, identification and management of patients at risk of progression is necessary to further improve outcomes. Our data suggest that a proportion of patients with persistent stable disease post-ICI may have viable residual tumor in those lesions. They also suggest that high FDG-avidity can be present in lesions with and without viable tumor cells, and low FDG-avidity in cases of viable (and proliferative) tumor nests, suggesting that FDG-avidity is neither specific (e.g., may represent uptake fludeoxyglucose-18 uptake by proliferating non-malignant cells) or sensitive (e.g., with a small lesion) in identifying patients who would benefit from a biopsy. The presence of reservoirs of viable and actively dividing tumor cells in stable lesions is likely to have valuable implications for clinical monitoring and patient management. However, it is noteworthy that patients in our cohort with detected viable tumor cells did not progress in eight years of subsequent follow-up, including the patient who only underwent biopsy (specimen MEL105) without complete resection of the residual lesion.

This study is necessarily limited by the relatively small number of patients, largely due to the challenge of identifying individuals who had persistent lesions amenable to safe biopsy and enrolling them in a research study (rather than following standard of care). We are nonetheless able to detect two different outcomes for ICI therapy: one in which tumors are actively dividing and dying, and the second in which all tumor cells are eradicated, and no residual disease is present. These two outcomes were not readily distinguishable by FDG-PET imaging alone, suggesting the potential value of biopsying persistent tumors. Further study of tumor mass dormancy, as opposed to tumor cell quiescence, is clearly warranted in human specimens and we encourage careful analysis of those persistent stable tumors that are biopsied or resected.

## METHODS

### Clinical Samples

Patient samples from a recent publication (Buchbinder et al) or from patients with a medical history of melanoma progression after ICI treatment (Supplementary Table 1) were retrieved from the archives of the Department of Pathology at Brigham and Women’s Hospital under BWH Institutional Review Board approval protocol 05–042 under a waiver of consent. Following de-identification, spatial profiling of these specimens is approved under HMS Institutional Review Board Protocol IRB21–0656. Fresh 5 μm sections were cut from each tumor block. The first section of each block was H&E stained for histopathological review and annotation of the sample. The remaining sections were characterized using immunohistochemistry (IHC), cyclic immunofluorescence (CyCIF, ORION) microscopy and microregional whole-transcriptome profiling (Nanostring GeoMx).

Treatment history and clinical metadata is shown in Supplementary Table 1. In brief, patient **MEL101** was diagnosed with metastatic melanoma in 2013, with lesions in the brain, left axilla, supraclavicular lymph nodes, and adrenal glands. The patient received four cycles of ipilimumab (anti-CTLA-4 blockade), resulting in a response and resolution of all lesions except for a persistent residual lymph node metastasis in the left axilla. At the time of this analysis (17 years post-initiation of ipilimumab), the patient remains free of recurrent disease. The clinical courses for patients **MEL102–MEL106** are described in the previous publication.(5) Patient **MEL107** was diagnosed with metastatic melanoma involving the lungs and initially treated with pembrolizumab without clinical response. Subsequent administration of two doses of ipilimumab resulted in a complete response. Eighteen months later, a recurrent right axillary lymph node metastasis was resected and included in this study. Patient **MEL108** received initial treatment consisting of surgical resection and a tumor vaccine. One year later, the patient experienced recurrence and was treated with ipilimumab and nivolumab, yielding a mixed response. A non-responding left axillary lymph node was resected and included in this study. The patient ultimately died of melanoma five years after the initial diagnosis. Patient **MEL109** presented with nodal metastatic melanoma and underwent cervical lymph node resection followed by ipilimumab. The patient progressed 4 months later and was started on pembrolizumab, which was subsequently held due to pneumonitis. Ten months after initiating pembrolizumab, she again showed disease progression and was re-started on pembrolizumab, achieving a complete response and completing two years of therapy. Ten months after stopping pembrolizumab, she developed progression in an inguinal lymph node, and the biopsy of that node was used for this study.

### Tissue imaging (H&E, IHC, CyCIF)

H&E and IHC staining of FFPE sections was performed by the Brigham and Women’s Hospital (BWH) Pathology Core and digitized using an Olympus VS-120 automated microscope using a 20x/0.75 NA objective at the Neurobiology Imaging Core at Harvard Medical School. IHC for cleaved caspase-3 used rabbit monoclonal antibody (clone A5E1, 1:500 dilution, Cell Signaling Technologies) followed by HRP-based chromogenic detection and hematoxylin counterstain. CyCIF was performed for specimens MEL101 and MEL107–MEL109 as previously described(18) and at protocols.io (dx.doi.org/10.17504/protocols.io.j8nlkoqbdv5r/v1). In brief, the BOND RX automated slide stainer was used to bake slides at 60°C for 30 minutes, dewax using Bond Dewax solution at 72°C and perform using Epitope Retrieval 1 (Leica) solution at 100°C for 20 minutes. After 4 rounds of photochemical bleaching to reduce tissue autofluorescence, slides underwent multiple cycles of antibody incubation, imaging, and fluorophore inactivation. Tissues were incubated overnight in the dark at 4°C in antibody solution containing Hoechst. Coverslips were mounted with 70% glycerol 1x PBS prior to imaging with a CyteFinder slide-scanning fluorescence microscope (RareCyte Inc.) with a 20x/0.75 NA objective. Slides were soaked in 42°C PBS for facilitate coverslip removal, and then fluorophores were inactivated by photochemical bleaching in a tray containing 4.5% H2O2 in PBS supplemented with 24 mM NaOH placed on a LED light source for one hour at room temperature. The list of all the antibodies and their dilutions for each experiment is presented in Supplemental Table 3.

### Tissue imaging (Orion)

Tissue imaging with RareCyte ORION platform (RRID:SCR_026855) was performed for specimens MEL102–MEL106 with following protocol previously described by Lin et al.(19) In brief, tissue sections were deparaffinized and subjected to heat-induced epitope retrieval. Slides were incubated at 95 °C for 5 min and then at 105 °C for 5 min in EZ-AR2 Elegance buffer (BioGenex HX032). Autofluorescence was reduced by treatment with a peroxide-based quenching solution (4.5% H_2_O_2_, 25 mM NaOH in PBS) under illumination, followed by washes in surfactant-containing buffer. Slides were then incubated in ORION Signal Enhancer for 15 min, washed, and stained overnight at 4 °C with the multiplex antibody panel. After primary staining, sections were washed and incubated for 30 min with anti-DIG antibody and streptavidin to detect DIG- and biotin-labeled probes. Slides were washed and coverslipped with ORION mounting medium (RareCyte WA). For sequential H&E staining, ORION-stained sections were decoverslipped in PBS for overnight, followed by the standard H&E protocol.

### CyCIF and ORION image pre-processing and quality control

Assembly of raw CyCIF and ORION imaging data into a high-dimensional images, as well as nuclear segmentation and single-cell feature extraction, were performed using the open-source MCMICRO(43) processing pipeline (RRID:SCR_022832), an open-source multiple-choice microscopy pipeline (version:38182748aa0ec021f684ce47248c57340d2f4cc7; full codes available at https://github.com/labsyspharm/mcmicro). The specific parameters used were optimized after iterative inspection of results, specifically focused on performance of the segmentation module to ensure accurate identification of single cells (params.yml files available at https://github.com/labsyspharm/2025_Shi_Exceptional_Responder_Manuscript ). After generating the segmentation masks, the mean fluorescence intensities of each marker for each cell were computed, resulting in a single-cell data table for each acquired whole-slide CyCIF and ORION image. The X/Y coordinates of annotated histologic regions on the whole slide image were used to extract the single-cell data of cells that lie within the ROI range. Multiple approaches were also taken to ensure the quality of the single-cell data. At the image level, the cross-cycle image registration and tissue integrity were reviewed; regions that were poorly registered or contained severely deformed tissues and artifacts were identified, and cells inside those regions were excluded. Antibodies that gave low confidence staining patterns by visual evaluation were also excluded from the analyses. The quality of the segmentation was assessed, and the segmentation parameters were iteratively modified to improve the accuracy of the segmentation masks.

### CyCIF and ORION single-cell gating-based phenotyping

Cells from CyCIF and ORION data were phenotyped using a gating-based classification approach as previously described.(44) In brief, gates for each marker were defined using an open-source visualization tool (https://github.com/labsyspharm/gater). These gates were then used to rescale single-cell expression values between 0 and 1, with values above 0.5 indicating marker positivity. The normalized data were subsequently used for cell-type calling and unsupervised clustering. Phenotype labels were assigned using the SCIMAP(45) Python package (RRID:SCR_024751) based on a hierarchical classification of marker expression patterns (Supplementary Fig. 2a). Assigned cell types were validated by overlaying phenotype labels onto the corresponding images.

### Definition of microanatomical regions

Microanatomical regions (tumor, peritumoral, necrotic, and perivascular) were defined using the SpatialCells package (https://github.com/SemenovLab/SpatialCells).(46) Single-cell centroid coordinates of SOX10^+^ tumor cells were used to delineate tumor boundaries by applying density-based spatial clustering (DBSCAN) followed by polygon-based boundary reconstruction to defined contiguous tumor regions. Regions immediately adjacent to tumor boundaries were classified as peritumoral regions. Necrotic regions were subclassified within peritumoral region based on the presence of DNA debris staining and sparse cell counts. Perivascular regions were defined as areas proximal to CD31^+^ endothelial cells. After region assignment, cell-type proportion within each microanatomical regions (shown in Supplementary Fig. 3d) were calculated by normalizing the number of cells of each cell type to the total number of that cell type across Domains A–E.

### Clustering and UMAP (CyCIF data)

To define tumor cell states from CyCIF imaging data, SOX10^+^ tumor cells were filtered from the full dataset MEL101A Level 1, Slide 2 and analyzed using the SCIMAP(45) package in Python. Expression intensity of all the protein markers was normalized based on gating thresholds and used to construct a k-nearest neighbor graph. Dimensionality reduction was performed using UMAP, and unsupervised clustering was conducted using the Leiden clustering with a resolution of 0.3 to identify distinct tumor clusters.

### Multivariate Proliferation Index (MPI) calculation

The Multivariate Proliferation Index (MPI) was based on the normalized measurement of five markers: three proliferation markers (KI67, CCNA2, CCNB1) and two cell-cycle arrest markers (p21, p27). The method avoids relying on single markers while separating cells expressing high level arrest markers (even if proliferation markers are expressed). The detailed methodology can be found in Gaglia, G. et al.(21)

### CyCIF kernel density estimation and contour mapping

For each phenotypic population, spatial density maps were generated using kernel density estimation (KDE) on single-cell centroid coordinates. All cells within each field were plotted as low-opacity background points to provide spatial context. For each phenotype, cells were selected and, when the number of cells was at least 50, a Gaussian KDE was applied to the centroid coordinates of marker positive cells with intensity clipped at the 98^th^ percentile and regions below the 10^th^ percentile masked for contrast enhancement. Density values were evaluated at cell positions and interpolated onto a regular two-dimensional grid to generate continuous density surfaces. Upper density values were clipped, and lower-density regions were masked and colored according to KDE-derived density intensity.

### CyCIF neighborhood spatial analysis

Cell centroid coordinates were converted to microns. A Delaunay triangulation was performed on the coordinates of the target cell population, and edges were adaptively filtered based on local nearest-neighbor distance to retain short-range connections. Cells located within 50 μm of any target cell were defined as neighbors. In Fig. 2c (perivascular region), CD8^+^ T cells and CD31^+^ endothelial cells were highlighted, and the filtered CD31^+^ connectivity network was visualized as line segments.

### CyCIF proximity analysis

CyCIF proximity analyses were performed using the *spatial_distance* function in the SCIMAP python package.(45) For each single cell in the CyCIF data, the average distance to specific cell phenotypes or clusters was calculated in two-dimensional coordinate space, enabling quantitative assessment of spatial relationships among cell populations within tissue sections.

### Microregion transcriptomics (GeoMx^®^) sample processing, data collection, and annotation

Digital spatial transcriptomic profiling was performed using the NanoString GeoMx^®^ Human Whole Transcriptome Atlas (WTA) RNA probes, following previously published protocols.(47,48) Briefly, freshly cut (< 2 weeks old) 5-μm thick FFPE melanoma sections were baked at 60 °C for 3 hours, dewaxed, and hybridized overnight with WTA probes. The following day, slides were incubated with fluorescence-conjugated antibodies targeting melanocytes (SOX10, 1:100, Abcam ab270151; MART1, 1:200, Abcam ab225500), and immune cells (CD45) before imaging and transcript collection on DSP (Supplementary Fig. 2c, Supplementary Table S3). These fluorescent marker signals were integrated with histopathology guides for MR selection. A total of 236 MRs were selected to present morphologically distinct zones, including the tumor edge, tumor center, peritumoral stroma, and tertiary lymphoid structure. Collected MRs were pooled for downstream library preparation and transcriptomic analysis.

### GeoMx analysis

GeoMx RNA sequencing data were processed using the Bioconductor GeoMxTools RNA-NGS workflow (https://www.bioconductor.org/packages/release/workflows/vignettes/GeoMxWorkflows/inst/doc/GeomxTools_RNA-NGS_Analysis.html). Raw count data were imported, quality controlled, and normalized according to the recommended pipeline, including probe-level filtering and sample-level quality control. Normalized expression matrices were used to perform t-SNE dimensionality reduction to visualize MR-level transcriptional heterogeneity.

### GeoMx gene signature analysis

Gene signature analysis was performed using DESeq2-normalized counts (obtained vis *counts(dds, normalized = TRUE)*) followed up log10 transformation. Single-sample gene set enrichment analysis (ssGSEA) scores were calculated using the GSVA R package (https://bioconductor.org/packages/release/bioc/GSVA/; RRID:SCR_021058), and pathway activity scores were used for downstream comparative analyses (e.g., Fig. 3i). Pathways included G1/S,(48) G2/M,(48) APOPTOSIS (Hallmark),(49) NECROPTOSIS (GO:BP),(50) PYROPTOSIS [Reactome],(51) FERRORPTSIS (WP),(52) and QUIESCENCE.(27)

### Single-cell RNA-seq analysis

Single-cell RNA-seq data was generated using 10x Genomics Chromium platform and processed with Cell Ranger(53) (RRID:SCR_017344) for alignment, demultiplexing, and gene-cell count matrix generation. Downstream analyses were performed in Python using Scanpy(54) (RRID:SCR_018139). Cells with low UMI counts, low gene detection, or high mitochondrial transcript content were filtered, and genes expressed in only few cells were removed. Data were normalized, log-transformed, and highly variable genes were selected. Dimensionality reduction was performed using PCA, followed by neighborhood graph construction, UMAP embedding, and Leiden clustering. Clusters were annotated into major cell types based on canonical marker gene expression.

### RNA velocity analysis

RNA velocity was computed from spliced and unspliced read counts generated using Velocyto(55) (RRID:SCR_018167). Downstream analysis was performed with the scVelo package(56) (RRID:SCR_018168). Moments were calculated on the k-nearest neighbor graph, velocities were estimated using the dynamic model, and a velocity graph was constructed. Velocity streamlines were projected onto the UMAP embedding and visualized over cell-type labels to infer transcriptional trajectories, with particular focus on state transitions within CD8^+^ T cells subsets.

### Single-cell RNA differential expression and gene set enrichment analysis

Differential gene expression of tumor cell clusters in Fig. 3c was performed using *rank_genes_groups* function from Scanpy.(54) For tumor cluster 4, the top 100 upregulated genes ranked by log fold change were selected for downstream enrichment analysis. Functional enrichment was performed using gene ontology–based pathway analysis, and enrichment scores were calculated. Dot plots were generated to visualize significantly enriched biological processes, where dot size represents the percentage of genes in each gene set and color indicates −log10-transformed p-values.

### Statistical Tests

All statistical comparisons between groups were performed using the Mann-Whitney U rank test to assess differences in the distribution of values between groups, implemented via the *mannwhitneyu* function in the SciPy(57) Python package (RRID: SCR_008058) or the stats and ggpubr R packages (RRID:SCR_021139).

## Supplementary Material

Supplement 1

Supplement 2

Supplement 3

Supplement 4

Supplement 5

## Data Availability

GeoMx gene expression data will be made available via the Gene Expression Omnibus (GEO; RRID: SCR_005012). All image data supporting the findings of this study are available via an index page on GitHub (https://github.com/labsyspharm/2025_Shi_Exceptional_Responder_Manuscript), which has been archived on Zenodo (http://doi.org/10.5281/zenodo.17807597). Code used for multimodal spatial analysis is available on GitHub.

## SIGNIFICANCE

This study demonstrates that, following immunotherapy, persistent stable melanomas can arise through tumor mass dormancy rather than quiescence. Mass dormancy is characterized by active tumor cell proliferation and compensatory programmed cell death. Thus, viable tumor cells in some stable lesions have the potential to reactivate disease should ongoing immunosurveillance fail.

## References

[1] van Not OJ, van den Eertwegh AJM, Jalving H, Bloem M, Haanen JB, van Rijn RS, Long-Term Survival in Patients With Advanced Melanoma. JAMA Netw Open. 2024;7:e2426641. 39141388 10.1001/jamanetworkopen.2024.26641 PMC11325208

[2] Wolchok JD, Chiarion-Sileni V, Rutkowski P, Cowey CL, Schadendorf D, Wagstaff J, Final, 10-Year Outcomes with Nivolumab plus Ipilimumab in Advanced Melanoma. N Engl J Med. Massachusetts Medical Society; 2025;392:11–22. 39282897 10.1056/NEJMoa2407417 PMC12080919

[3] Noringriis IM, Donia M, Madsen K, Schmidt H, Haslund CA, Bastholt L, Long-term clinical outcome of patients with metastatic melanoma and initial stable disease during anti-PD-1 checkpoint inhibitor immunotherapy with pembrolizumab. Br J Cancer. Nature Publishing Group; 2025;133:337–45. 40419744 10.1038/s41416-025-03048-8 PMC12322104

[4] Prieto PA, Yang JC, Sherry RM, Hughes MS, Kammula US, White DE, CTLA-4 Blockade with Ipilimumab: Long-Term Follow-up of 177 Patients with Metastatic Melanoma. Clin Cancer Res. 2012;18:2039–47. 22271879 10.1158/1078-0432.CCR-11-1823 PMC3319861

[5] Buchbinder EI, Pfaff KL, Turner MM, Manos M, Ouyang O, Ott PA, Brief Communication on Pathologic Assessment of Persistent Stable Metastatic Lesions in Patients Treated With Anti-CTLA-4 or Anti-CTLA-4 + Anti-PD-1 Therapy. J Immunother Hagerstown Md 1997. 2023;46:192–6. 10.1097/CJI.0000000000000470 PMC10168111 37115942

[6] Endo H, Inoue M. Dormancy in cancer. Cancer Sci. 2019;110:474–80. 30575231 10.1111/cas.13917 PMC6361606

[7] Holmgren L, O’Reilly MS, Folkman J. Dormancy of micrometastases: balanced proliferation and apoptosis in the presence of angiogenesis suppression. Nat Med. 1995;1:149–53. 7585012 10.1038/nm0295-149

[8] Mills CE, Subramanian K, Hafner M, Niepel M, Gerosa L, Chung M, Multiplexed and reproducible high content screening of live and fixed cells using Dye Drop. Nat Commun. 2022;13:6918. 36376301 10.1038/s41467-022-34536-7 PMC9663587

[9] Koebel CM, Vermi W, Swann JB, Zerafa N, Rodig SJ, Old LJ, Adaptive immunity maintains occult cancer in an equilibrium state. Nature. 2007;450:903–7. 18026089 10.1038/nature06309

[10] Risson E, Nobre AR, Maguer-Satta V, Aguirre-Ghiso JA. The current paradigm and challenges ahead for the dormancy of disseminated tumor cells. Nat Cancer. Nature Publishing Group; 2020;1:672–80. 33681821 10.1038/s43018-020-0088-5 PMC7929485

[11] Giancotti FG. Mechanisms Governing Metastatic Dormancy and Reactivation. Cell. 2013;155:750–64. 24209616 10.1016/j.cell.2013.10.029 PMC4354734

[12] Baldominos P, Barbera-Mourelle A, Barreiro O, Huang Y, Wight A, Cho J-W, Quiescent cancer cells resist T cell attack by forming an immunosuppressive niche. Cell. 2022;185:1694–1708.e19. 35447074 10.1016/j.cell.2022.03.033 PMC11332067

[13] Wikman H, Vessella R, Pan℡ K. Cancer micrometastasis and tumour dormancy. APMIS. 2008;116:754–70. 18834417 10.1111/j.1600-0463.2008.01033.x

[14] Ruth JR, Pant DK, Pan T-C, Seidel HE, Baksh SC, Keister BA, Cellular dormancy in minimal residual disease following targeted therapy. Breast Cancer Res BCR. 2021;23:63. 34088357 10.1186/s13058-021-01416-9 PMC8178846

[15] Schatton T, Murphy GF, Frank NY, Yamaura K, Waaga-Gasser AM, Gasser M, Identification of cells initiating human melanomas. Nature. Nature Publishing Group; 2008;451:345–9. 18202660 10.1038/nature06489 PMC3660705

[16] Quintana E, Shackleton M, Sabel MS, Fullen DR, Johnson TM, Morrison SJ. Efficient tumor formation by single human melanoma cells. Nature. 2008;456:593–8. 19052619 10.1038/nature07567 PMC2597380

[17] Hossain SM, Eccles MR. Phenotype Switching and the Melanoma Microenvironment; Impact on Immunotherapy and Drug Resistance. Int J Mol Sci. 2023;24:1601. 36675114 10.3390/ijms24021601 PMC9864717

[18] Lin J-R, Izar B, Wang S, Yapp C, Mei S, Shah PM, Highly multiplexed immunofluorescence imaging of human tissues and tumors using t-CyCIF and conventional optical microscopes. eLife. 2018;7:e31657. 29993362 10.7554/eLife.31657 PMC6075866

[19] Lin J-R, Chen Y-A, Campton D, Cooper J, Coy S, Yapp C, High-plex immunofluorescence imaging and traditional histology of the same tissue section for discovering image-based biomarkers. Nat Cancer. Nature Publishing Group; 2023;4:1036–52. 37349501 10.1038/s43018-023-00576-1 PMC10368530

[20] Tetzlaff MT, Messina JL, Stein JE, Xu X, Amaria RN, Blank CU, Pathological assessment of resection specimens after neoadjuvant therapy for metastatic melanoma. Ann Oncol Off J Eur Soc Med Oncol. 2018;29:1861–8. 10.1093/annonc/mdy226 PMC6096739 29945191

[21] Gaglia G, Kabraji S, Rammos D, Dai Y, Verma A, Wang S, Temporal and spatial topography of cell proliferation in cancer. Nat Cell Biol. 2022;24:316–26. 35292783 10.1038/s41556-022-00860-9 PMC8959396

[22] Liu Y, Pan R, Ouyang Y, Gu W, Xiao T, Yang H, Pyroptosis in health and disease: mechanisms, regulation and clinical perspective. Signal Transduct Target Ther. Nature Publishing Group; 2024;9:245. 39300122 10.1038/s41392-024-01958-2 PMC11413206

[23] Meier P, Legrand AJ, Adam D, Silke J. Immunogenic cell death in cancer: targeting necroptosis to induce antitumour immunity. Nat Rev Cancer. Nature Publishing Group; 2024;24:299–315. 38454135 10.1038/s41568-024-00674-x

[24] Chen W, He Y, Zhou G, Chen X, Ye Y, Zhang G, Multiomics characterization of pyroptosis in the tumor microenvironment and therapeutic relevance in metastatic melanoma. BMC Med. 2024;22:24. 38229080 10.1186/s12916-023-03175-0 PMC10792919

[25] Wiernicki B, Maschalidi S, Pinney J, Adjemian S, Vanden Berghe T, Ravichandran KS, Cancer cells dying from ferroptosis impede dendritic cell-mediated anti-tumor immunity. Nat Commun. 2022;13:3676. 35760796 10.1038/s41467-022-31218-2 PMC9237053

[26] Bagaev A, Kotlov N, Nomie K, Svekolkin V, Gafurov A, Isaeva O, Conserved pan-cancer microenvironment subtypes predict response to immunotherapy. Cancer Cell. 2021;39:845–865.e7. 34019806 10.1016/j.ccell.2021.04.014

[27] Xie XP, Laks DR, Sun D, Ganbold M, Wang Z, Pedraza AM, Quiescent human glioblastoma cancer stem cells drive tumor initiation, expansion, and recurrence following chemotherapy. Dev Cell. 2022;57:32–46.e8. 35016005 10.1016/j.devcel.2021.12.007 PMC8820651

[28] Bresnick AR, Weber DJ, Zimmer DB. S100 proteins in cancer. Nat Rev Cancer. Nature Publishing Group; 2015;15:96–109. 25614008 10.1038/nrc3893 PMC4369764

[29] Sviatoha V, Tani E, Kleina R, Sperga M, Skoog L. Immunohistochemical analysis of the S100A1, S100B, CD44 and Bcl-2 antigens and the rate of cell proliferation assessed by Ki-67 antibody in benign and malignant melanocytic tumours. Melanoma Res. 2010;20:118–25. 20042890 10.1097/CMR.0b013e3283350554

[30] Koch A, Lang SA, Wild PJ, Gantner S, Mahli A, Spanier G, Glucose transporter isoform 1 expression enhances metastasis of malignant melanoma cells. Oncotarget. 2015;6:32748–60. 26293674 10.18632/oncotarget.4977 PMC4741727

[31] Cheng Y, Li J, Martinka M, Li G. The expression of NAD(P)H:quinone oxidoreductase 1 is increased along with NF-kappaB p105/p50 in human cutaneous melanomas. Oncol Rep. 2010;23:973–9. 20204281 10.3892/or_00000722

[32] Widmer DS, Hoek KS, Cheng PF, Eichhoff OM, Biedermann T, Raaijmakers MIG, Hypoxia contributes to melanoma heterogeneity by triggering HIF1α-dependent phenotype switching. J Invest Dermatol. 2013;133:2436–43. 23474946 10.1038/jid.2013.115

[33] Mahmoud A, Ganesh K. Mouse Models of Metastasis and Dormancy. Cold Spring Harb Perspect Med. 2024;14:a041386. 37696656 10.1101/cshperspect.a041386 PMC10925556

[34] Liu Z, Jiao D. Necroptosis, tumor necrosis and tumorigenesis. Cell Stress. 4:1–8. 31922095 10.15698/cst2020.01.208 PMC6946014

[35] Ladstein RG, Bachmann IM, Straume O, Akslen LA. Tumor necrosis is a prognostic factor in thick cutaneous melanoma. Am J Surg Pathol. 2012;36:1477–82. 22982891 10.1097/PAS.0b013e31825a5b45

[36] Lo SN, Scolyer RA, Thompson JF. Long-Term Survival of Patients with Thin (T1) Cutaneous Melanomas: A Breslow Thickness Cut Point of 0.8 mm Separates Higher-Risk and Lower-Risk Tumors. Ann Surg Oncol. 2018;25:894–902. 10.1245/s10434-017-6325-1 29330716

[37] Strauss DC, Thomas JM. Transmission of donor melanoma by organ transplantation. Lancet Oncol. 2010;11:790–6. 20451456 10.1016/S1470-2045(10)70024-3

[38] Penn I. Malignant melanoma in organ allograft recipients. Transplantation. 1996;61:274–8. 8600636 10.1097/00007890-199601270-00019

[39] Eyles J, Puaux A-L, Wang X, Toh B, Prakash C, Hong M, Tumor cells disseminate early, but immunosurveillance limits metastatic outgrowth, in a mouse model of melanoma. J Clin Invest. American Society for Clinical Investigation; 2010;120:2030–9. 20501944 10.1172/JCI42002 PMC2877955

[40] Wolchok JD, Chiarion-Sileni V, Gonzalez R, Rutkowski P, Grob J-J, Cowey CL, Overall Survival with Combined Nivolumab and Ipilimumab in Advanced Melanoma. N Engl J Med. Massachusetts Medical Society; 2017;377:1345–56. 28889792 10.1056/NEJMoa1709684 PMC5706778

[41] Handel EE, McKeown J, Wei J, Kankaria RA, Burnette H, Johnson DB, Outcomes following long-term disease control with immune checkpoint inhibitors in patients with advanced melanoma. Eur J Cancer. 2025;215:115171. 39667250 10.1016/j.ejca.2024.115171

[42] Perez L, Samlowski W, Lopez-Flores R. Outcome of Elective Checkpoint Inhibitor Discontinuation in Patients with Metastatic Melanoma Who Achieved a Complete Remission: Real-World Data. Biomedicines. 2022;10:1144. 35625881 10.3390/biomedicines10051144 PMC9138966

[43] Schapiro D, Sokolov A, Yapp C, Chen Y-A, Muhlich JL, Hess J, MCMICRO: a scalable, modular image-processing pipeline for multiplexed tissue imaging. Nat Methods. 2022;19:311–5. 34824477 10.1038/s41592-021-01308-y PMC8916956

[44] Nirmal AJ, Maliga Z, Vallius T, Quattrochi B, Chen AA, Jacobson CA, The Spatial Landscape of Progression and Immunoediting in Primary Melanoma at Single-Cell Resolution. Cancer Discov. 2022;12:1518–41. 35404441 10.1158/2159-8290.CD-21-1357 PMC9167783

[45] Nirmal AJ, Sorger PK. SCIMAP: A Python Toolkit for Integrated Spatial Analysis of Multiplexed Imaging Data. J Open Source Softw. 2024;9:6604. 38873023 10.21105/joss.06604 PMC11173324

[46] Wan G, Maliga Z, Yan B, Vallius T, Shi Y, Khattab S, SpatialCells: automated profiling of tumor microenvironments with spatially resolved multiplexed single-cell data. Brief Bioinform. 2024;25:bbae189. 38701421 10.1093/bib/bbae189 PMC11066940

[47] Zollinger DR, Lingle SE, Sorg K, Beechem JM, Merritt CR. GeoMx^™^ RNA Assay: High Multiplex, Digital, Spatial Analysis of RNA in FFPE Tissue. Methods Mol Biol Clifton NJ. 2020;2148:331–45. 10.1007/978-1-0716-0623-0_21 32394392

[48] Jerby-Arnon L, Shah P, Cuoco MS, Rodman C, Su M-J, Melms JC, A Cancer Cell Program Promotes T Cell Exclusion and Resistance to Checkpoint Blockade. Cell. 2018;175:984–997.e24. 30388455 10.1016/j.cell.2018.09.006 PMC6410377

[49] Liberzon A, Birger C, Thorvaldsdóttir H, Ghandi M, Mesirov JP, Tamayo P. The Molecular Signatures Database (MSigDB) hallmark gene set collection. Cell Syst. 2015;1:417–25. 26771021 10.1016/j.cels.2015.12.004 PMC4707969

[50] Gene Ontology Consortium. The Gene Ontology resource: enriching a GOld mine. Nucleic Acids Res. 2021;49:D325–34. 33290552 10.1093/nar/gkaa1113 PMC7779012

[51] Jassal B, Matthews L, Viteri G, Gong C, Lorente P, Fabregat A, The reactome pathway knowledgebase. Nucleic Acids Res. 2020;48:D498–503. 31691815 10.1093/nar/gkz1031 PMC7145712

[52] Martens M, Ammar A, Riutta A, Waagmeester A, Slenter DN, Hanspers K, WikiPathways: connecting communities. Nucleic Acids Res. 2021;49:D613–21. 33211851 10.1093/nar/gkaa1024 PMC7779061

[53] Zheng GXY, Terry JM, Belgrader P, Ryvkin P, Bent ZW, Wilson R, Massively parallel digital transcriptional profiling of single cells. Nat Commun. 2017;8:14049. 28091601 10.1038/ncomms14049 PMC5241818

[54] Wolf FA, Angerer P, Theis FJ. SCANPY: large-scale single-cell gene expression data analysis. Genome Biol. 2018;19:15. 29409532 10.1186/s13059-017-1382-0 PMC5802054

[55] La Manno G, Soldatov R, Zeisel A, Braun E, Hochgerner H, Petukhov V, RNA velocity of single cells. Nature. 2018;560:494–8. 30089906 10.1038/s41586-018-0414-6 PMC6130801

[56] Bergen V, Lange M, Peidli S, Wolf FA, Theis FJ. Generalizing RNA velocity to transient cell states through dynamical modeling. Nat Biotechnol. 2020;38:1408–14. 32747759 10.1038/s41587-020-0591-3

[57] Virtanen P, Gommers R, Oliphant TE, Haberland M, Reddy T, Cournapeau D, SciPy 1.0: fundamental algorithms for scientific computing in Python. Nat Methods. 2020;17:261–72. 32015543 10.1038/s41592-019-0686-2 PMC7056644

